# Association between the composite dietary antioxidant index and cardiovascular-kidney-metabolic syndrome among U.S. adults: evidence from NHANES 2007–2018

**DOI:** 10.3389/fnut.2025.1600651

**Published:** 2025-07-10

**Authors:** Qiuming He, Fan Hu, Wanhui Wei, Jie Li, Yang Yu, Heng Zhang

**Affiliations:** ^1^Department of Gastroenterology, The Central Hospital of Wuhan, Tongji Medical College, Huazhong University of Science and Technology, Wuhan, China; ^2^Key Laboratory for Molecular Diagnosis of Hubei Province, The Central Hospital of Wuhan, Tongji Medical College, Huazhong University of Science and Technology, Wuhan, China; ^3^Department of Cardiology, The Central Hospital of Wuhan, Tongji Medical College, Huazhong University of Science and Technology, Wuhan, China

**Keywords:** CDAI, CKM, nutrition, oxidative stress, NHANES

## Abstract

**Objective:**

Cardiovascular-kidney-metabolic (CKM) syndrome is a major public health issue worldwide. However, direct evidence on dietary modulators in CKM syndrome is lacking. This study aimed to explore the association between the Composite Dietary Antioxidant Index (CDAI) and advanced CKM syndrome using National Health and Nutrition Examination Survey (NHANES) from 2007 to 2018.

**Methods:**

Advanced CKM syndrome (Stage 3–4) was defined using 2023 AHA criteria. CDAI was calculated from averaged 24-h dietary recalls for six antioxidants (vitamins A/C/E, zinc, selenium, carotenoids). Weighted multivariable logistic regression adjusted for sociodemographics, lifestyle, and metabolic factors. Multinomial logistic regression was used to estimate odds ratios (ORs) and 95% confidence intervals (CI), adjusting for potential confounders. Furthermore, restricted cubic splines (RCS) were applied to investigate any possible nonlinear relationships between CDAI and CKM syndrome in the study.

**Results:**

This study included 11,073 adults aged 20 years and older, with a mean age of 48 years and a gender distribution of 52.75% female and 47.25% male. Multivariate logistic regression with full adjustment for covariates showed that higher CDAI scores were inversely associated with advanced CKM syndrome. Specifically, compared to the lowest quartile, the highest quartile of CDAI scores had an OR of 0.70 (95% CI: 0.49–0.98). A nonlinear negative correlation was identified by the RCS (*p* for nonlinearity = 0.031). In both the subgroup and sensitivity analysis, this relationship was still present.

**Conclusion:**

Higher CDAI scores are correlated with decreased odds of advanced CKM syndrome, suggesting that an antioxidant-rich diet may be associated with a lower likelihood of advanced CKM syndrome. Understanding these correlations could contribute to the development of preventive strategies and intervention measures for CKM syndrome. However, prospective studies are needed to confirm these associations and explore their clinical relevance.

## Introduction

Cardiovascular-kidney-metabolic (CKM) syndrome imposes a great burden on society due to its significant impact on morbidity and mortality, as defined by the AHA in October 2023 (1, 2). CKM syndrome is recognized as a health disorder characterized by the interrelationships among cardiovascular disease, renal disease, diabetes, and obesity, which collectively contribute to adverse health outcomes. This syndrome heightens the risk for both the onset and advancement of cardiovascular disease, encompassing individuals who are at risk as well as those who already have established cardiovascular conditions (2). CKM syndrome highlights the complex interplay among metabolic disorders, chronic kidney disease (CKD), and cardiovascular disease (CVD) (3), an interplay which exacerbates disease progression and worsens cardiovascular outcomes (4). To characterize CKM syndrome progression, the AHA proposed a staging system (0–4), with advancing stages correlating with increased risks of multiorgan dysfunction and adverse cardiovascular events (4). Due to aging populations and lifestyle factors, the number of people with CKM syndrome is increasing. Thus, early identification and comprehensive management are essential to reducing its clinical and public health burden.

Oxidative stress is a core process and central component in the pathophysiology of CKM syndrome, driving the progression of organ damage and dysfunction (5). Hyperglycemia, impaired cardiac and renal function, and metabolic disturbances contribute to elevated oxidative stress through mechanisms such as the activation of the renin-angiotensin-aldosterone system, increased production of reactive oxygen species (ROS), and the formation of advanced glycation end products and protein kinase C activation (6). The above processes accelerate inflammation, endothelial dysfunction, and tissue damage, which exacerbate CKM syndrome-related complications (7). Given the critical role of oxidative stress in CKM syndrome, assessing oxidative status is essential for effective risk stratification, staging, and predicting outcomes in CKM syndrome patients.

Dietary antioxidants play a crucial role in modulating oxidative stress and inflammation, key contributors to chronic disease progression (8). The CDAI is a validated tool that quantifies overall dietary antioxidant intake, incorporating key micronutrients such as vitamins A, C, and E, carotenoid, zinc, and selenium (9). These antioxidants exert anti-inflammatory effects by reducing pro-inflammatory mediators, including tumor necrosis factor-*α* (TNF-α) and interleukin-1β (IL-1β) (10). Higher CDAI scores have been associated with lower risks of hypertension, CKD, and diabetes, as well as improved outcomes in ocular diseases such as age-related macular degeneration (AMD) and glaucoma (11–14). Despite growing evidence supporting the protective effects of antioxidant-rich diets, the role of CDAI in CKM syndrome remains underexplored.

In this study, we used data from the NHANES 2007–2018. We aimed to discover the potential association between CDAI and CKM syndrome, with the goal to inform dietary recommendations for CKM syndrome prevention.

## Materials and methods

### Sources of information

This study utilized data from NHANES database. NHANES is a cross-sectional study conducted by the National Center for Health Statistics (NCHS) under the Centers for Disease Control and Prevention to assess the health and nutritional status of the U. S. population (15). This comprehensive survey employs household and telephone interviews, as well as physical examinations, to provide an extensive dataset reflecting the health status of the U. S. population. NHANES employs a complex multistage probability sampling design to ensure national representativeness. The NCHS Research Ethics Review Board approved the study protocol, and all participants provided written informed consent (16). Data are publicly available on the NHANES website at https://www.cdc.gov/nchs/nhanes/index.htm.

### Study population

**Figure 1:**
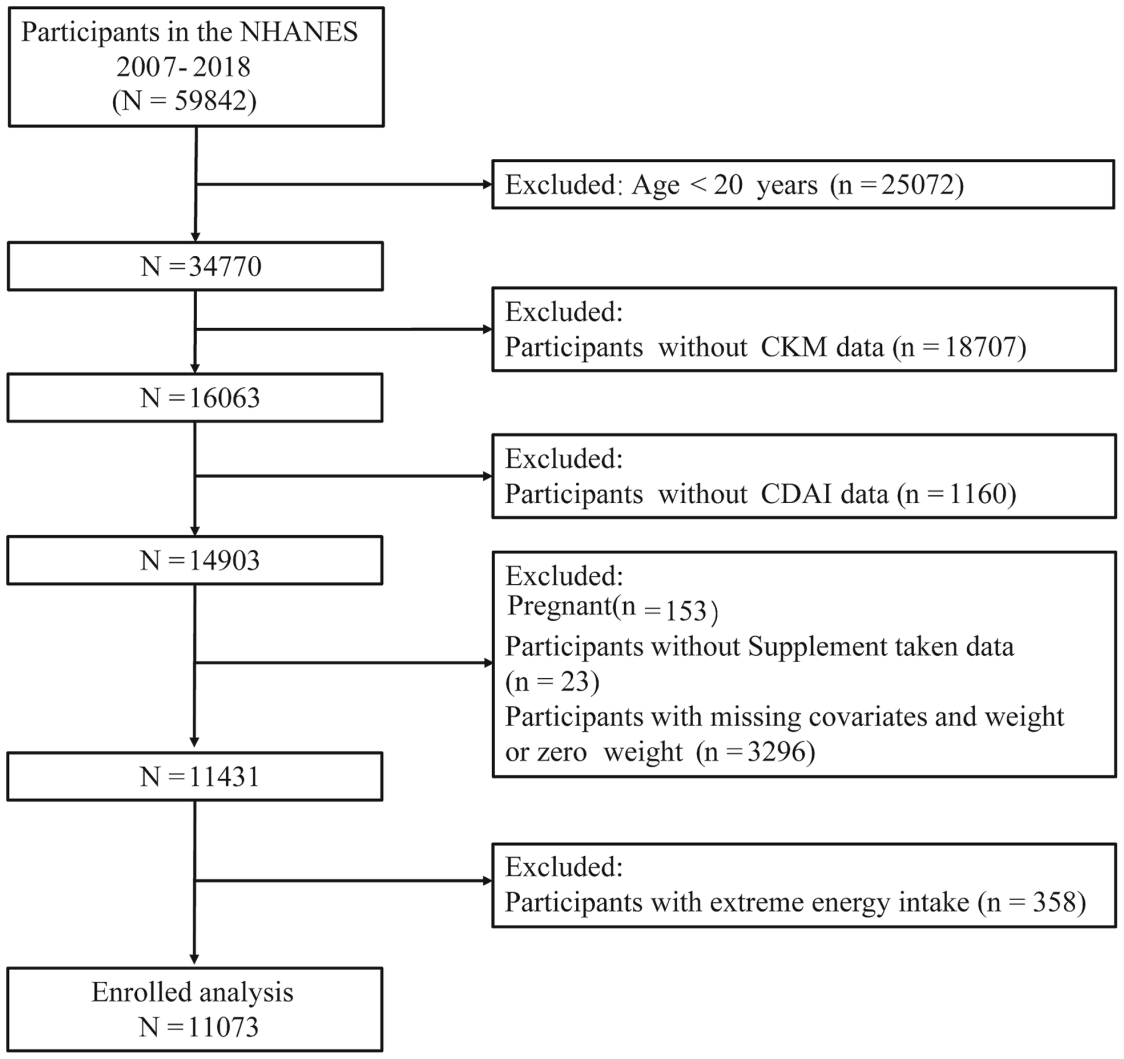
Flowchart of participant selection process.

In this study, data were selected from eight NHANES survey cycles spanning from 2007 to 2018. Data collection methods included questionnaires, interview records, physical examinations, and laboratory tests. To ensure national representativeness and valid variance estimation, all analyses incorporated the appropriate 12-year sample weights, strata, and primary sampling units (PSUs), as recommended by NHANES analytic guidelines. Initially, the study included 59,842 participants from consecutive NHANES datasets. The following exclusion criteria were applied: (1) participants under 20 years of age (*n* = 25,072); (2) participants lacking CKM syndrome data (*n* = 18,707); (3) participants missing CDAI component data (*n* = 1,160); (4) pregnant participants (*n* = 153); (5) participants without supplement taken data (*n* = 23) and with missing relevant covariates (*n* = 3,296) and (6) participants with extreme energy intake (*n* = 358). After a rigorous data screening process, a total of 11,073 participants were selected for further analysis. A comprehensive flowchart illustrating the participant selection process is presented in Figure 1.

### Definition of CKM syndrome

CKM syndrome is recognized as a health disorder characterized by the interrelationships among cardiovascular disease, renal disease, diabetes, and obesity, which collectively contribute to adverse health outcomes. According to the 2023 American Heart Association (AHA) scientific statement, CKM syndrome encompasses overlapping dysfunction across these systems, driven by shared mechanisms including inflammation, oxidative stress, and metabolic dysregulation (1). In this study, we classified participants into five stages of CKM syndrome (Stage 0 to Stage 4) in accordance with the 2023 American Heart Association (AHA) statement and operationalized the criteria using variables available in the NHANES database (17). Detailed staging definitions and variable mappings are provided in Supplementary Table S1.

Stage 0: Individuals at this stage are distinguished by the lack of CKM risk factors, such as hypertension. Defined as having no apparent cardiometabolic risk factors. Participants in this category had normal body mass index (BMI < 25 kg/m^2^), normal waist circumference (< 88 cm for females, < 102 cm for males), normoglycemia (fasting plasma glucose [FBG] < 100 mg/dL and HbA1c < 5.7%), normotension (SBP < 130 mmHg and DBP < 80 mmHg), and normal lipid levels (triglycerides < 135 mg/dL), without evidence of CKD or CVD.

Stage 1: Individuals who were overweight or obese (BMI ≥ 25 kg/m^2^), increased waist circumference (≥ 88 cm for females, ≥ 102 cm for males), or prediabetes (HbA1c 5.7–6.4% or FBG 100–125 mg/dL), but without metabolic risk factors or CKD.

Stage 2: Participants were identified as having metabolic risk factors or moderate-to-high-risk chronic kidney disease (CKD) in accordance with the Kidney Disease: Improving Global Outcomes (KDIGO) guidelines. The metabolic risk factors encompassed elevated triglyceride levels (≥135 mg/dL), hypertension, diabetes, or metabolic syndrome ≥ 3 of the following: increased waist circumference, low high-density lipoprotein (HDL) levels (< 40 mg/dL for males and < 50 mg/dL for females), elevated triglyceride levels (≥150 mg/dL), elevated blood pressure (SBP ≥ 130 mmHg or DBP ≥ 80 mmHg), or prediabetes.

Stage 3: Participants were assigned to this stage if they met criteria for very high-risk CKD (eGFR < 30 mL/min/1.73m^2^ or KDIGO-classified very high risk) or had a high predicted 10-year cardiovascular risk (≥ 20%), as estimated by the 2024 AHA PREVENT equations (18). These equations, implemented via the AHA PREVENT Risk Calculator [https://professional.heart.org/en/guidelines-and-statements/prevent-calculator], incorporate demographic, clinical, and laboratory variables to assess 10-year CVD risk.

Stage 4: Defined as a self-reported history of coronary heart disease, angina pectoris, myocardial infarction, congestive heart failure, or stroke. Due to data limitations, conditions such as atrial fibrillation or peripheral arterial disease were not included. According to the prevalence of CKM syndrome stages in US adults, advanced CKM syndrome was defined as Stage 3 or Stage 4, representing individuals who either currently exhibit or are at an elevated risk of developing cardiovascular disease (CVD) (19).

### Assessment of CDAI

Dietary intake data were collected using 24-h dietary recalls conducted at mobile examination centers. These data were converted into nutrient intake values using the U. S. Department of Agriculture’s Food and Nutrient Database for Dietary Studies. For each participant, dietary intake was assessed as the average of two separate 24-h dietary recall records (20). For participants with both recalls available, we used the average of the two days.

The CDAI is a composite score incorporating various dietary antioxidants, including vitamins A, C, E, selenium, zinc, and carotenoids. The CDAI was calculated using the method described by Wright et al., which involves subtracting the population mean intake of each dietary antioxidant from the individual’s intake and then dividing the result by the population standard deviation (20). CDAI is a standardized composite index based on z-scores of six dietary antioxidants. Positive CDAI values indicate above-average total dietary antioxidant intake, while negative values indicate below-average intake relative to the population mean. The sum of these values constitutes the CDAI, as expressed by the following formula:


$$\text{CDAI}=\sum_{i=1}^{6}\frac{\text{Individual Intake}-\text{Mean}}{\mathrm{SD}}.$$

To maintain the integrity and dependability of the data, participants exhibiting abnormal total energy intake (> 4,200 or < 800 kcal/day in males; > 3,500 or < 500 kcal/day in females) were excluded from the analysis.

### Data collection

Demographic data, physical examination results, laboratory measurements, lifestyle factors, and medical conditions were collected. Demographic data: Age, sex, race, and education level. Physical examination: Body mass index (BMI), systolic blood pressure (SBP), and diastolic blood pressure (DBP). Laboratory tests: Serum creatinine (Scr), glycated hemoglobin (HbA1c), total cholesterol (TC), high-density lipoprotein cholesterol (HDL-C), estimated glomerular filtration rate (eGFR, mL/min/1.73m^2^), and urinary albumin-to-creatinine ratio (UACR, mg/g). Self-reported questionnaires: Information on smoking status, alcohol consumption, diabetes, and hypertension history. BMI was calculated as weight (kg) divided by height squared (m^2^). BMI ≤ 25 was classified as normal weight, 25–30 as overweight, and ≥ 30 as obesity. SBP and DBP were computed as the mean of four measurements. Smoking status was categorized as never, former, or current smoker. Alcohol consumption was classified into five categories: never, former, light, moderate, and heavy. Hypertension was defined as SBP ≥ 140 mmHg, DBP ≥ 80 mmHg, a medical diagnosis, or antihypertensive medication use. Diabetes was characterized by fasting blood glucose (FBG) levels ≥ 126 mg/dL (7.0 mmol/L), HbA1c levels ≥ 6.5%, a medical diagnosis, or the use of insulin or glucose-lowering medications. Hyperlipidemia was defined comprehensively, and participants were classified as having hyperlipidemia if they met any one of the following criteria: triglycerides ≥ 150 mg/dL, total cholesterol ≥ 200 mg/dL, LDL-C ≥ 130 mg/dL, HDL-C < 40 mg/dL (male) or < 50 mg/dL (female), or current use of lipid-lowering medication. eGFR was calculated using the 2021 race-free Chronic Kidney Disease Epidemiology Collaboration (CKD-EPI) creatinine equation. CKD was classified into low, moderate, high, and very high risk based on the Kidney Disease: Improving Global Outcomes (KDIGO) guidelines using eGFR and UACR (21).

### Statistical analysis

To account for the complex multistage sampling design of NHANES, all analyses incorporated survey design factors, including sample weights, clustering, and stratification, to ensure nationally representative estimates. Data analysis was conducted using R version 4.4.1 with the “nhanesR” package. Continuous and categorical variables were presented as mean (95%CI) and frequency (weighted percentage), respectively. Weighted *t*-tests were used for comparisons of continuous variables, while survey-weighted chi-square tests were employed for categorical variables.

Weighted univariate and multivariate logistic regression analyses were performed to explore the association between CDAI and advanced CKM syndrome. Three models were constructed: Model 1: Unadjusted. Model 2: Adjusted for age, gender, race, and education levels. Model 3: Adjusted for age, gender, race, education levels, marital status, alcohol intake, energy intake, supplement use.

Restricted cubic spline (RCS) analysis was performed to examine potential nonlinear relationships between CDAI and advanced CKM syndrome. Subgroup analyses were conducted based on age, race, education levels, marital status, alcohol intake, energy intake and supplement use to assess the stability of the association between CDAI and advanced CKM syndrome across different subgroups. A *p*-value of less than 0.05 was considered statistically significant.

## Results

### Baseline characteristics of study participants stratified by CDAI quartiles

The baseline characteristics of participants stratified by CDAI quartiles are presented in Table 1. A total of 11,073 participants were included in the analysis, with a mean age of 48 years. Of these, 52.75% were female and 47.25% were male. The overall weighted prevalence of advanced CKM syndrome in the total sample was 13.30%. Notably, participants in higher CDAI quartiles exhibited a lower unadjusted prevalence of advanced CKM syndrome within each quartile (Q1: 28.49%; Q2: 25.64%; Q3: 24.84%; Q4: 21.03%). Significant differences (*p* < 0.05) were observed across CDAI quartiles in terms of gender, race/ethnicity, education levels, alcohol intake, energy intake, supplement use, and the prevalence of diabetes, hyperlipidemia, cardiovascular disease, and chronic kidney disease prognosis.

**Table 1:** Characteristics of participants stratified by the CDAI quartiles.

Characteristics	Total (*N* = 11,073)	Quartiles of composite dietary antioxidant index (CDAI)				*P* value
		Q1 (*N* = 2,770)	Q2 (*N* = 2,767)	Q3 (*N* = 2,768)	Q4 (*N* = 2,768)	
		< −2.214	−2.214 to −0.231	−0.231 to 2.231	> 2.231	
Age	48.00 (34.00,61.00)	48.00 (33.00,62.00)	48.00 (34.00,63.00)	49.00 (34.00,61.00)	48.00 (34.00,59.00)	0.28
Sex						0.004
Male	5,233 (47.25)	1,217 (42.39)	1,337 (47.71)	1,358 (48.70)	1,321 (49.41)	
Female	5,840 (52.75)	1,553 (57.61)	1,430 (52.29)	1,410 (51.30)	1,447 (50.59)	
Race						< 0.0001
White	4,863 (67.25)	1,142 (63.80)	1,200 (65.33)	1,271 (69.70)	1,250 (69.30)	
Black	2,158 (10.16)	650 (13.18)	537 (10.42)	494 (9.33)	477 (8.27)	
Mexican American	1,641 (8.43)	396 (8.55)	404 (8.94)	410 (8.12)	431 (8.18)	
Other Hispanic	1,193 (5.93)	312 (6.81)	325 (6.35)	281 (4.87)	275 (5.89)	
Other	1,218 (8.24)	270 (7.67)	301 (8.95)	312 (7.98)	335 (8.36)	
Education levels						< 0.0001
Less than high school	1,029 (5.09)	348 (7.42)	283 (5.79)	213 (4.47)	185 (3.19)	
High school or equivalent	3,969 (32.77)	1,196 (42.19)	1,043 (34.35)	895 (30.41)	835 (26.06)	
College or above	6,075 (62.14)	1,226 (50.40)	1,441 (59.85)	1,660 (65.13)	1748 (70.75)	
BMI (kg/m2)	27.90 (24.10,32.60)	27.80 (24.00,32.70)	28.20 (24.50,33.00)	28.22 (24.06,32.50)	27.50 (24.02,32.30)	0.14
BMI (kg/m^2^)						0.4
normal	3,135 (30.50)	775 (30.62)	723 (28.17)	789 (30.71)	848 (32.40)	
obesity	4,281 (37.49)	1,088 (38.40)	1,102 (39.67)	1,057 (37.37)	1,034 (35.25)	
overweight	3,632 (31.82)	896 (30.98)	937 (32.16)	915 (31.92)	884 (32.35)	
Scr (mg/dL)	0.83 (0.71,0.98)	0.84 (0.71,0.99)	0.84 (0.72,0.99)	0.83 (0.71,0.97)	0.84 (0.71,0.96)	0.64
HbA1c	5.50 (5.20,5.80)	5.50 (5.20,5.80)	5.50 (5.20,5.80)	5.40 (5.20,5.80)	5.40 (5.20,5.70)	0.12
TC (mg/dL)	189.00 (164.00,217.00)	188.00 (162.00,218.00)	193.00 (167.00,221.00)	187.00 (164.00,215.00)	189.00 (163.00,214.00)	0.005
HDL-C (mg/dL)	52.00 (43.00,63.00)	51.00 (42.00,62.00)	51.00 (43.00,61.00)	52.00 (43.00,64.00)	52.00 (43.00,64.00)	0.03
UACR (mg/g)	6.50 (4.29,11.80)	7.64 (4.74,14.22)	6.49 (4.25,11.79)	6.39 (4.13,11.40)	6.05 (4.17,10.55)	< 0.0001
eGFR (ml/min/1.73 m2)	98.09 (83.62, 110.73)	97.25 (81.40, 110.81)	97.51 (81.40, 109.85)	97.85 (84.89, 110.41)	99.00 (85.57, 111.79)	0.03
SBP (mmHg)	119.00 (110.00,130.00)	120.00 (109.00,132.00)	119.00 (110.00,130.00)	119.00 (110.00,129.00)	118.00 (109.00,129.00)	0.01
DBP (mmHg)	70.00 (63.00,77.00)	69.00 (61.00,76.00)	70.00 (62.00,76.00)	69.00 (63.00,76.00)	71.00 (64.00,78.00)	0.002
Hypertension						0.08
No	6,285 (61.50)	1,460 (58.34)	1,552 (60.73)	1,626 (63.21)	1,647 (63.07)	
Yes	4,788 (38.50)	1,310 (41.66)	1,215 (39.27)	1,142 (36.79)	1,121 (36.93)	
DM						0.01
No	8,713 (84.00)	2,115 (81.53)	2,125 (83.06)	2,207 (84.39)	2,266 (86.41)	
Yes	2,360 (16.00)	655 (18.47)	642 (16.94)	561 (15.61)	502 (13.59)	
Hyperlipidemia						0.004
No	3,004 (28.50)	670 (25.05)	709 (26.76)	790 (31.03)	835 (30.33)	
Yes	8,069 (71.50)	2,100 (74.95)	2058 (73.24)	1978 (68.97)	1933 (69.67)	
CVD						< 0.001
No	9,834 (90.82)	2,356 (87.84)	2,447 (90.03)	2,495 (91.69)	2,536 (93.06)	
Yes	1,238 (9.18)	414 (12.16)	320 (9.97)	272 (8.31)	232 (6.94)	
CKD prognosis						< 0.0001
low risk	9,233 (87.10)	2,197 (82.28)	2,283 (87.13)	2,361 (88.34)	2,392 (89.80)	
moderate risk	1,273 (9.39)	368 (12.04)	339 (9.45)	286 (8.69)	280 (7.85)	
high risk	350 (2.35)	114 (3.49)	91 (2.17)	75 (2.02)	70 (1.88)	
very high risk	217 (1.17)	91 (2.19)	54 (1.25)	46 (0.96)	26 (0.47)	
Mets						0.24
No	6,817 (62.96)	1,669 (61.15)	1,682 (61.45)	1738 (64.79)	1728 (63.95)	
Yes	4,256 (37.04)	1,101 (38.85)	1,085 (38.55)	1,030 (35.21)	1,040 (36.05)	
CDAI	0.12 (−1.97, 2.58)	−3.28 (−4.13, −2.71)	−1.17 (−1.68, −0.71)	0.83 (0.27, 1.50)	4.48 (3.31, 6.64)	< 0.0001
Energy intake (kcal/day)	1973.50 (1536.50, 2500.50)	1480.50 (1161.00,1889.00)	1848.50 (1489.50, 2223.50)	2107.00 (1736.50, 2580.50)	2415.50 (1920.00,2965.50)	< 0.0001
Alcohol intake (g/day)						< 0.001
>30	881 (9.71)	201 (8.85)	205 (8.91)	232 (11.23)	243 (9.76)	
0	7,887 (67.14)	2096 (73.44)	2017 (69.71)	1910 (63.56)	1864 (64.26)	
0.1–30	2,258 (22.75)	464 (17.71)	534 (21.38)	611 (25.21)	649 (25.98)	
Supplement use						< 0.0001
No	6,139 (54.23)	1711 (61.92)	1,558 (55.43)	1,468 (53.01)	1,402 (48.14)	
Yes	4,934 (45.77)	1,059 (38.08)	1,209 (44.57)	1,300 (46.99)	1,366 (51.86)	
CKM						< 0.001
No	11,271 (86.70)	2,624 (20.89)	2,799 (23.58)	2,868 (26.85)	2,980 (28.68)	
Yes	2,447 (13.30)	808 (28.42)	628 (24.88)	561 (25.39)	450 (21.31)	

BMI, body mass index; Scr, serum creatinine; TC, total cholesterol; HDL-C, HDL cholesterol; UACR, urine albumin to creatinine ratio; eGFR, estimated glomerular filtration rate; SBP, systolic blood pressure; DBP, diastolic blood pressure; CVD, cardiovascular disease; CKD, chronic kidney disease prognosis; CDAI, composite dietary antioxidant index.

CDAI is a standardized composite index based on *z*-scores of six dietary antioxidants. Positive CDAI values indicate above-average total dietary antioxidant intake, while negative values indicate below-average intake relative to the population mean.

Compared to participants in the lowest CDAI quartile, those with higher CDAI values were more likely to be younger, female, non-Hispanic White, have higher education levels, no alcohol intake, higher energy intake and with supplement use. Regarding biochemical and physiological parameters, participants in the highest CDAI quartile exhibited significantly lower levels of HbA1c, TC, UACR and SBP, while eGFR and DBP were significantly higher. Further analysis indicated that the prevalence of chronic diseases such as hypertension, diabetes, hyperlipidemia, CVD, and CKD prognosis was significantly lower in the highest CDAI quartile compared to the lowest.

### Baseline characteristics of study participants stratified by CKM syndrome stages

The baseline characteristics of participants stratified by CKM syndrome stages were shown in Table S2. The mean CDAI index observed in the study was 0.12. Compared to participants in the early CKM syndrome stage, those in the advanced CKM syndrome stage exhibited distinctive characteristics, including older age, male, non-Hispanic White, and relatively lower educational attainment.

Additionally, participants in the advanced CKM syndrome stage demonstrated significant alterations in physiological parameters. Specifically, they exhibited increased BMI, SBP, Scr, HbA1c, and UACR, whereas DBP, TC, HDL-C, eGFR, alcohol intake and energy intake were decreased.

### Association between CDAI and advanced CKM syndrome

Table 2 presents the results of weighted logistic regression analysis examining the association between CDAI and advanced CKM syndrome. In Models 1, 2, and 3, treating CDAI as a continuous variable revealed a significant negative association with advanced CKM syndrome [Model 1: OR = 0.94, 95% CI (0.91, 0.96), *p* < 0.001; Model 2: OR = 0.95, 95% CI (0.93, 0.98); *p* < 0.001; Model 3: OR = 0.96, 95% CI (0.94, 0.99), *p* = 0.02]. Further quartile analysis indicated that, compared to the lowest quartile, the highest quartile of CDAI exhibited a significant negative association with advanced CKM syndrome in Models 1 and 2. This association persisted in Model 3 after adjusting for all covariates [Q4, Model 3: OR = 0.70, *p* = 0.04, 95% CI (0.49, 0.98)]. Trend tests across all models reinforced this association (Model 3: *p* for trend = 0.04), suggesting that participants with higher CDAI values had a significantly reduced risk of advanced CKM syndrome.

**Table 2:** Association between CDAI and advanced CKM Syndrome: Multivariable Logistic Regression Results.

	Model 1		Model 2		Model 3	
Characteristics	OR (95%CI)	*P*	OR (95%CI)	*P*	OR (95%CI)	*P*
CDAI	0.94 (0.91,0.96)	<0.0001	0.95 (0.93,0.98)	<0.001	0.96 (0.94,0.99)	0.02
Quartile of CDAI						
Q1	Reference		Reference		Reference	
Q2	0.84 (0.68,1.03)	0.09	0.74 (0.57,0.95)	0.02	0.78 (0.60,1.00)	0.05
Q3	0.69 (0.56,0.87)	0.001	0.65 (0.49,0.85)	0.002	0.72 (0.53,0.97)	0.03
Q4	0.56 (0.44,0.71)	<0.0001	0.61 (0.46,0.82)	0.001	0.70 (0.49,0.98)	0.04
*p* for trend		<0.0001		<0.001		0.04

Model 1: unadjusted model.

Model 2: adjusted for age, gender, race and education levels.

Model 3: adjusted for age, gender, race, education levels, marital status, alcohol intake, energy intake, supplement use.

**Figure 2:**
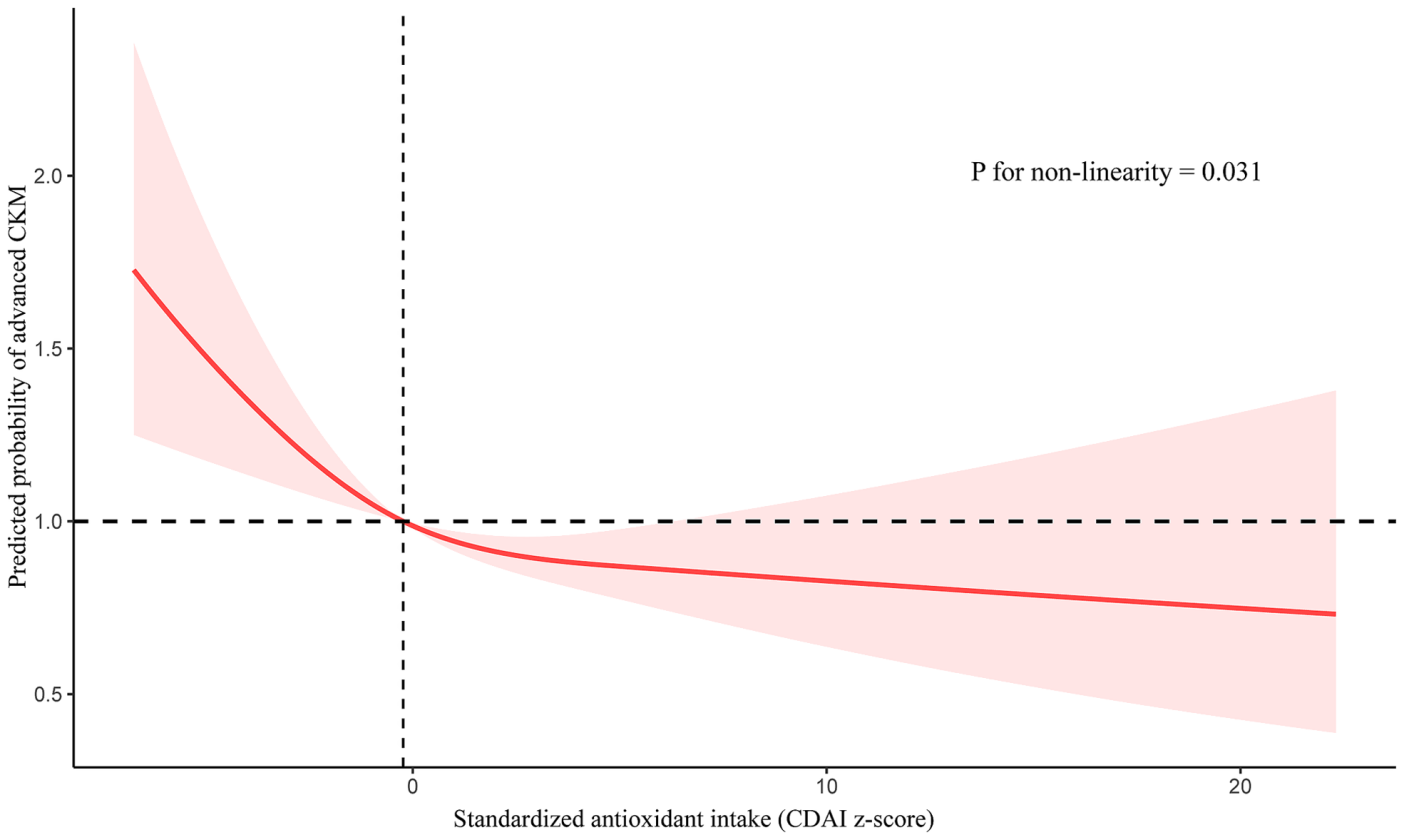
RCS for the relationship between CDAI and advanced CKM syndrome.

RCS analysis demonstrated a significant nonlinear trend in the association between CDAI and advanced CKM syndrome (*p* for nonlinearity = 0.031), suggesting a complex relationship beyond a simple linear association (Figure 2). At lower CDAI levels, the risk of advanced CKM syndrome declined with increasing CDAI values, whereas at higher CDAI levels, this trend plateaued.

### Association between six antioxidants of CDAI components and advanced CKM syndrome

Subsequent weighted logistic regression analyses were performed to assess the independent effects of the six antioxidant components of CDAI on advanced CKM syndrome (Table 3). When treated as continuous variables, only vitamin E showed a significant inverse association with advanced CKM syndrome in the fully adjusted model, whereas no significant associations were observed for the other individual antioxidants. When antioxidant intake was categorized into quartiles, several antioxidants demonstrated dose-dependent relationships. Notably, higher quartiles of vitamin A, vitamin C, vitamin E, zinc, and carotenoids intake were all associated with lower odds of advanced CKM syndrome to varying degrees. These findings suggest that specific antioxidant nutrients may offer protective effects against advanced stages of cardiometabolic-kidney dysfunction.

**Table 3:** The weighted logistic regression analysis of the association between six components of CDAI and advanced CKM syndrome.

	Model 1		Model 2		Model 3	
Components	OR (95%CI)	*P*	OR (95%CI)	*P*	OR (95%CI)	*P*
Vitamin A (μg/day)						
Continuous	1.00 (1.00,1.00)	0.24	1.00 (1.00,1.00)	0.01	1.00 (1.00,1.00)	0.06
Q1	Reference		Reference		Reference	
Q2	1.03 (0.83,1.28)	0.78	0.81 (0.62,1.07)	0.14	0.87 (0.66,1.14)	0.30
Q3	1.08 (0.84,1.39)	0.55	0.76 (0.55,1.04)	0.08	0.83 (0.60,1.15)	0.26
Q4	0.95 (0.74,1.22)	0.70	0.69 (0.52,0.92)	0.01	0.80 (0.59,1.10)	0.17
*p* for trend		0.77		0.02		0.21
Vitamin C (mg/day)						
Continuous	1.00 (1.00,1.00)	0.26	1.00 (1.00,1.00)	0.01	1.00 (1.00,1.00)	0.10
Q1	Reference		Reference		Reference	
Q2	1.16 (0.93,1.46)	0.18	0.89 (0.65,1.21)	0.46	0.97 (0.71,1.33)	0.85
Q3	1.05 (0.86,1.30)	0.62	0.68 (0.52,0.89)	0.01	0.79 (0.60,1.05)	0.10
Q4	1.02 (0.80,1.28)	0.90	0.68 (0.51,0.89)	0.01	0.79 (0.59,1.07)	0.12
*p* for trend		0.87		<0.001		0.05
Vitamin E (mg/day)						
Continuous	0.97 (0.95,0.98)	<0.0001	0.97 (0.95,0.99)	<0.001	0.98 (0.96,0.99)	0.01
Q1	Reference		Reference		Reference	
Q2	0.75 (0.62,0.90)	0.003	0.74 (0.57,0.97)	0.03	0.79 (0.59,1.05)	0.10
Q3	0.62 (0.49,0.77)	<0.0001	0.55 (0.42,0.70)	<0.0001	0.60 (0.45,0.80)	<0.001
Q4	0.63 (0.50,0.80)	<0.001	0.66 (0.49,0.89)	0.01	0.77 (0.54,1.08)	0.13
*p* for trend		<0.001		0.003		0.06
Zinc (mg/day)						
Continuous	0.98 (0.96,0.99)	0.01	0.99 (0.97,1.01)	0.33	1.00 (0.98,1.02)	0.86
Q1	Reference		Reference		Reference	
Q2	0.87 (0.70,1.08)	0.20	0.93 (0.72,1.20)	0.58	1.04 (0.81,1.36)	0.74
Q3	0.69 (0.55,0.88)	0.003	0.77 (0.60,0.99)	0.04	0.91 (0.68,1.20)	0.49
Q4	0.68 (0.53,0.87)	0.003	0.82 (0.63,1.08)	0.15	1.03 (0.76,1.40)	0.85
*p* for trend		0.001		0.07		0.89
Selenium (μg/day)						
Continuous	1.00 (0.99,1.00)	<0.0001	1.00 (1.00,1.00)	0.11	1.00 (1.00,1.00)	0.48
Q1	Reference		Reference		Reference	
Q2	0.79 (0.62,1.00)	0.05	0.87 (0.67,1.13)	0.30	0.96 (0.74,1.25)	0.76
Q3	0.66 (0.53,0.82)	<0.001	0.83 (0.64,1.08)	0.16	0.97 (0.75,1.25)	0.80
Q4	0.56 (0.43,0.73)	<0.0001	0.86 (0.64,1.17)	0.33	1.03 (0.75,1.41)	0.85
*p* for trend		<0.0001		0.31		0.88
Carotenoid (μg/day)						
Continuous	1.00 (1.00,1.00)	0.01	1.00 (1.00,1.00)	0.03	1.00 (1.00,1.00)	0.13
Q1	Reference		Reference		Reference	
Q2	0.94 (0.75,1.19)	0.62	0.91 (0.69,1.19)	0.47	0.96 (0.73,1.27)	0.80
Q3	0.89 (0.71,1.12)	0.31	0.88 (0.66,1.16)	0.36	0.97 (0.72,1.30)	0.84
Q4	0.71 (0.58,0.87)	<0.001	0.68 (0.53,0.87)	0.002	0.77 (0.59,0.99)	0.05
*p* for trend		<0.001		0.003		0.06

**Figure 3:**
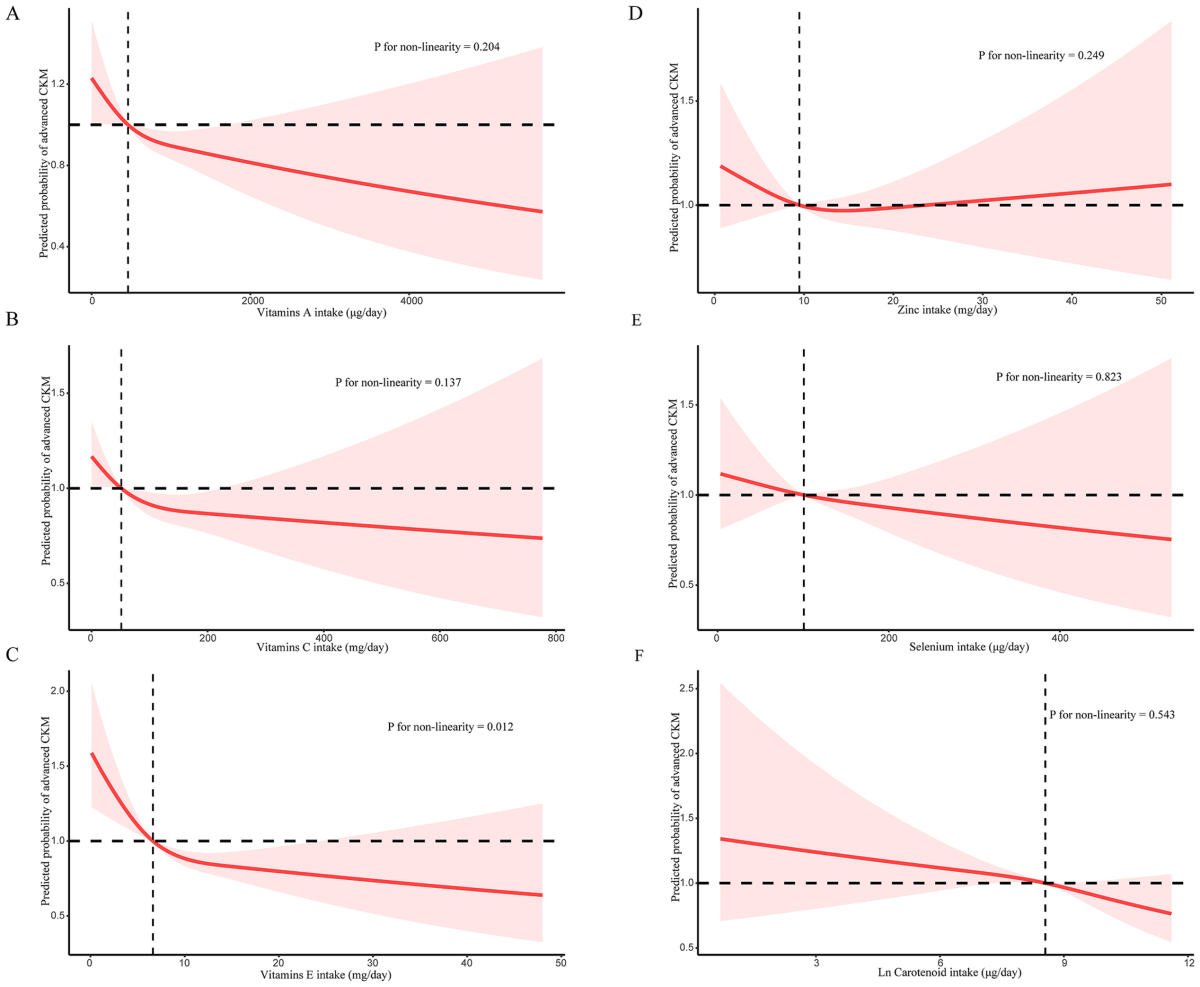
**(A)** RCS for the relationship between vitamin A and advanced CKM syndrome; **(B)** RCS for the relationship between vitamin C and advanced CKM syndrome; **(C)** RCS for the relationship between vitamin E and advanced CKM syndrome; **(D)** RCS for the relationship between zinc and advanced CKM syndrome; **(E)** RCS for the relationship between selenium and advanced CKM syndrome; **(F)** RCS for the relationship between carotenoid and advanced CKM syndrome.

Restricted cubic spline (RCS) analysis revealed a nonlinear, L-shaped association between vitamin E intake and advanced CKM syndrome (Figure 3). The probability of advanced CKM syndrome declined sharply with increasing vitamin E intake at lower intake levels, followed by a plateau at higher levels. This suggests that while adequate vitamin E consumption may offer protective benefits, additional intake beyond a certain point does not appear to confer further advantage. Due to the left-skewed distribution of carotenoids, the values were analyzed after natural logarithm transformation. Importantly, this nonlinear relationship was observed only for vitamin E; other antioxidant components in the CDAI—such as vitamin A, vitamin C, zinc, selenium, and carotenoids—showed linear or non-significant trends in the spline models. Taken together, these results emphasize that while dietary antioxidants may play a protective role in CKM syndrome, an optimal intake range likely exists, and higher doses may not be universally beneficial. This finding suggests a complex interplay between antioxidant levels and CKM syndrome.

### Subgroup analysis

**Figure 4:**
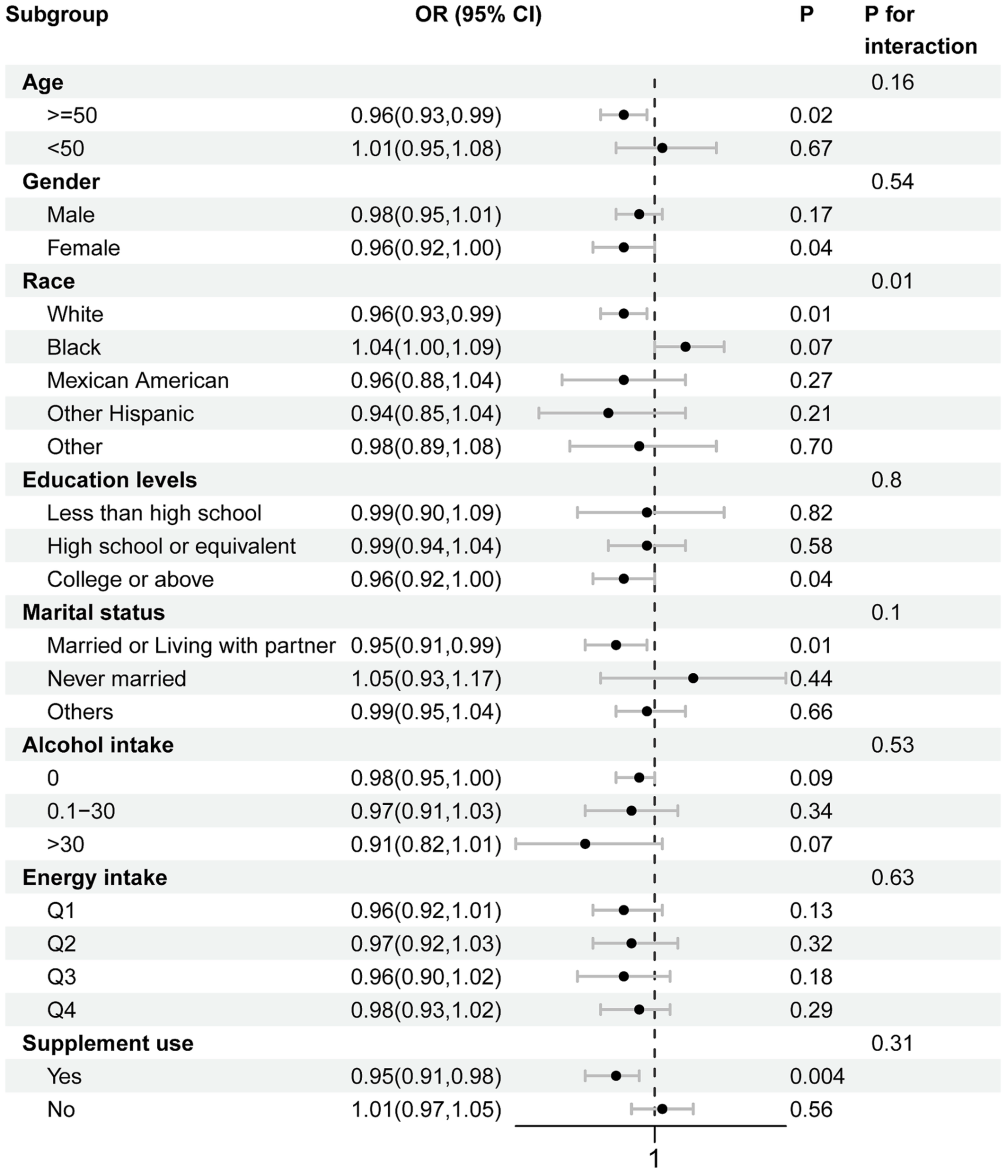
Subgroup analysis for relationship between CDAI and advanced CKM syndrome.

Subgroup analysis results are illustrated in Figure 4. The association of CDAI with advanced CKM syndrome remained consistent when stratifying by age, gender, educational levels, marital status, alcohol intake, energy intake and supplement use, with all *p* values for interaction being > 0.05. We observed a significant interaction between race and CDAI for advanced CKM syndrome (*P* interactio*n* = 0.01). Among individuals aged 50 years and above, a threshold chosen based on the mean age of the overall study population, CDAI was negatively associated with advanced CKM syndrome (OR = 0.96, 95% CI: 0.93–0.99, *p* = 0.02). A negative association was also observed in non-Hispanic White (OR = 0.96, 95% CI: 0.93–0.99, *p* = 0.01). Among individuals married or living with partner, CDAI was negatively associated with advanced CKM syndrome (OR = 0.95, 95% CI: 0.91–0.99, *p* = 0.01). Similarly, individuals with supplement use exhibited a negative association with advanced CKM syndrome (OR = 0.95, 95% CI: 0.91–0.98, *p* = 0.004).

## Discussion

In this research, we investigated the relationship between the Composite Dietary Antioxidant Index (CDAI) and advanced chronic kidney disease (CKM) syndrome within a nationally representative sample. Our findings revealed a significant inverse correlation, suggesting that increased dietary antioxidant consumption may be linked to a lower likelihood of advanced CKM syndrome. This relationship remained consistent after adjustment for confounders and across key subgroups, with stronger associations noted among individuals who were Mexican White and those with supplement use. These findings suggest a potential protective role of dietary antioxidants in CKM syndrome management and, to our knowledge, represent the first large-scale investigation of this association.

As a newly recognized clinical entity, CKM syndrome encompasses a complex interplay of metabolic, cardiovascular, and renal dysfunctions, making lifestyle interventions critical for its management (22–24). In our study, we defined advanced CKM syndrome by combining Stage 3 and Stage 4 based on the 2023 AHA scientific statement (1, 25). We recognize that these two stages may signify distinct positions within the continuum of cardiometabolic disease, with Stage 3 denoting a high predicted risk of cardiovascular disease (CVD) or very high-risk chronic kidney disease (CKD), and Stage 4 indicating the presence of overt cardiovascular or kidney disease. Although stages 3 and 4 of CKM syndrome represent distinct clinical statuses—stage 3 indicating elevated risk and stage 4 reflecting manifest disease—we opted to combine these stages into a single category termed ‘advanced CKM syndrome’ for several reasons. First, both stages represent individuals who are at substantially increased risk of adverse outcomes and are targeted for intensive clinical intervention under the unified care strategies proposed by the AHA. Second, due to the limited sample size within each individual stage in the NHANES dataset, combining them improved statistical power and ensured model robustness. Third, from a public health and nutritional epidemiology perspective, our goal was to assess whether overall antioxidant intake is associated with the risk of progressing to more severe disease states, regardless of whether risk is predicted or realized. We acknowledge the potential heterogeneity introduced by this approach, and we emphasize that our results warrant cautious interpretation. Future research should aim to assess the differential effects of dietary antioxidants on each individual CKM stage in longitudinal designs.

Dietary antioxidants, particularly carotenoids and vitamins A, C, and E, play a crucial role in modulating oxidative stress, a key factor associated with CKM syndrome (26–29). Lei Wu et al. reported that Carotenoids, abundant in fruits and vegetables, function as potent antioxidants and precursors to vitamin A, contributing to cellular protection and systemic homeostasis (30). CDAI, as a composite index of dietary antioxidants, has been associated with a variety of health outcomes, including rheumatoid arthritis (31), ocular diseases (32), non-alcoholic fatty liver disease (33) and hyperlipidemia (34). Our findings suggest that higher dietary antioxidant intake, as measured by the Composite Dietary Antioxidant Index (CDAI), is inversely associated with the likelihood of having advanced CKM syndrome consistent with prior research showing protective effects of antioxidants like carotenoids, vitamin C, and vitamin E against CVD (35), CKD (36), and metabolic dysfunction (37). This aligns with biological plausibility and extends previous research by using CDAI as a composite dietary index, providing a broader view of antioxidant exposure than studies focusing on individual nutrients. In the restricted cubic spline analysis, a statistically significant L-shaped association was observed, indicating a protective effect at low-to-moderate intakes that plateaued at higher levels. These results suggest that antioxidant benefits may reach a threshold, beyond which additional intake offers limited or no added advantage, consistent with the concept of biological optimum. Excessive intake, particularly from supplements, may even disturb redox homeostasis. Accordingly, while our findings support the protective potential of dietary antioxidants, they also underscore the importance of maintaining optimal, rather than maximal, intake levels. While two 24-h recalls may not capture habitual intake, this approach reduces random error and aligns with NHANES methodology. We also adjusted for total energy intake to reduce confounding by overall food consumption levels, which further supports the robustness of the CDAI-CKM syndrome association. Given that CKM syndrome involves oxidative stress, inflammation, and metabolic dysfunction, the observed inverse association may reflect the modulatory role of antioxidants in these pathways.

The stratified analysis conducted in this study indicated that the association between CDAI and CKM syndrome differed across age groups. Age is a critical modifier in the development of CKM syndrome, influencing both risk burden and physiological responses. With age, the accumulation of oxidative stress, mitochondrial dysfunction, vascular aging, and systemic inflammation accelerates, contributing to heightened susceptibility to cardiovascular, renal, and metabolic disorders. These age-related mechanisms may also amplify the potential protective effect of dietary antioxidants. Our subgroup finding—showing a stronger inverse association between CDAI and advanced CKM syndrome among individuals aged 50 years and older—supports this hypothesis. Furthermore, age 50 was selected as a stratification threshold because it reflects both the approximate mean age of our study population and a recognized turning point for cardiometabolic risk escalation. This choice ensured balanced group sizes and meaningful clinical interpretation.

CDAI is a dietary assessment tool that may have potential utility in informing risk assessment and preventive strategies for CKM syndrome. Monitoring CDAI levels could help identify individuals at higher likelihood of advanced CKM syndrome, enabling early dietary interventions aimed at enhancing antioxidant intake. Clinically, integrating CDAI assessments into routine evaluations may complement traditional metabolic and inflammatory markers, providing a more comprehensive approach to risk stratification. Future research should explore the incorporation of CDAI into CKM syndrome management strategies, with an emphasis on optimizing personalized dietary recommendations to mitigate disease progression and improve patient outcomes. As the CDAI was based only on food and beverage intake, we adjusted for dietary supplement use in all models to minimize potential misclassification among supplement users. The observed associations persisted after this adjustment, suggesting that the relationship between dietary antioxidants and CKM syndrome is not solely attributable to supplemental sources. Nonetheless, future research should consider integrating supplement-derived nutrients and non-nutrient antioxidants such as flavonoids or polyphenols to provide a more comprehensive assessment.

In this study, we examined the nonlinear association between the Composite Dietary Antioxidant Index (CDAI) and CKM syndrome, offering new insights into the potential protective role of dietary antioxidants. The use of a large, nationally representative sample and comprehensive statistical analyses strengthens the reliability, generalizability, and robustness of our findings. While previous studies have focused on individual antioxidants and their relationships with cardiovascular or renal diseases, our study provides a more comprehensive assessment using CDAI as a dietary index. This composite measure allows for a broader understanding of the dietary antioxidant intake’s role in CKM syndrome. Our research contributes to the existing body of literature by elucidating the intricate relationships among these health outcomes.

Several limitations should be acknowledged. First, due to the cross-sectional design of NHANES and lack of information on time of diagnosis, the determination of causal relationship between CDAI and CKM syndrome was prevented. It is plausible that individuals with CKM-related diagnoses may have modified their diets in response to medical advice or disease management strategies, thereby influencing their antioxidant intake levels. This potential reverse causation limits our ability to determine whether dietary antioxidants actively contribute to lower CKM risk or merely reflect behavior changes post-diagnosis. Second, although we adjusted for several key demographic and behavioral variables including age, gender, race/ethnicity, education, marital status, alcohol intake, energy intake, and supplement use, we cannot exclude the possibility of residual confounding from unmeasured healthy lifestyle factors, such as physical activity, sleep quality, or overall diet quality, which may partially account for the observed associations. Third, while our study focused on advanced CKM syndrome as a composite outcome aligned with the 2023 AHA framework, this approach does not allow us to determine which component—cardiovascular, renal, or metabolic—is most influenced by dietary antioxidants. Future research should consider evaluating specific endpoints, such as CKD progression or incident cardiovascular events, to clarify underlying biological mechanisms. Forth, CDAI was derived from one or two 24-h dietary recalls, which are subject to recall bias and day-to-day variation. Fifth, the operationalization of CKM syndrome using NHANES survey data may introduce misclassification bias, particularly when based on self-reported disease history or medication use. Moreover, selection bias might occur in the main analyses because a substantial number of participants were excluded due to predefined eligibility criteria or missing key data (e.g., dietary intake, supplement use, CKM syndrome staging variables). Furthermore, NHANES data were collected over a span of more than a decade (2007–2018), during which secular trends in clinical practice, diagnostics, or laboratory procedures may have introduced unmeasured cohort effects or heterogeneity. Lastly, it is essential to note that the findings of this study are specifically applicable to the population of the United States and cannot be generalized to other populations, necessitating further investigation. As the CKM syndrome framework is relatively new, further validation in international and prospective cohorts is needed to confirm its utility. While our findings are promising, the potential clinical application of CDAI remains hypothetical. Additional longitudinal and interventional studies are needed to evaluate its predictive value and role in risk stratification.

## Conclusion

In summary, our study revealed a significant inverse association between CDAI and advanced CKM syndrome, suggesting that a diet rich in antioxidants may be associated with a lower likelihood of advanced CKM syndrome. These findings highlight the potential clinical relevance of dietary antioxidant intake in reducing the burden of this multifactorial syndrome. Further prospective studies and interventional trials are needed to establish causality and explore the underlying mechanisms of this association, ultimately informing dietary recommendations for CKM syndrome prevention and management.

## Data Availability

The original contributions presented in the study are included in the article/Supplementary material, further inquiries can be directed to the corresponding authors.
