# Supplementary material for: Association between the composite dietary antioxidant index and cardiovascular-kidney-metabolic syndrome among U.S. adults: evidence from NHANES 2007–2018
Source: Front Nutr. 2025 Jul 10;12:1600651. doi: 10.3389/fnut.2025.1600651 (PMC12287050; doi:10.3389/fnut.2025.1600651)
Supplement: Supplementary file 1 [file Table_1.DOCX]

**Table S1**: Definitions of CKM stages using NHANES variables

| CKM Stage | Definition |
| --- | --- |
| CKM Stage 0 | - Participants with normal body mass index (BMI, < 25 kg/m²), waist circumference (<88 cm for women, < 102 cm for men), normoglycemia (fasting blood glucose [FBG] < 100 mg/dL, glycated hemoglobin [HbA1c] < 5.7%), normotension (systolic blood pressure [SBP] < 130 mmHg, diastolic blood pressure [DBP] < 80 mmHg), normal lipid profile (triglycerides < 135 mg/dL), and no evidence of chronic kidney disease (CKD) or clinical/subclinical cardiovascular disease (CVD). |
| CKM Stage 1 | - Participants with elevated BMI (≥ 25 kg/m²), increased waist circumference (≥ 88 cm for women, ≥ 102 cm for men), or prediabetes (HbA1c 5.7%-6.4% or FBG 100-125 mg/dL), without the presence of other metabolic risk factors or CKD. |
| CKM Stage 2 | - Participants with metabolic risk factors or moderate-to-high-risk CKD per KDIGO guidelines ^[1]^. Metabolic risk factors included elevated triglycerides (≥ 135 mg/dL), hypertension, diabetes, or metabolic syndrome (≥ 3 of the following: elevated waist circumference, low HDL [< 40 mg/dL for men, < 50 mg/dL for women], elevated triglycerides [≥ 150 mg/dL], elevated BP [systolic ≥ 130 mmHg, diastolic ≥ 80 mmHg], or prediabetes). |
| CKM Stage3 | - Participants with very-high-risk CKD (KDIGO criteria) or high 10-year CVD risk (≥20%) based on the AHA PREVENT equations ^[2]^. High risk was defined as ≥ 20% 10- year CVD risk (based on recommended thresholds [https://professional.heart.org/en/guidelines-and-statements/prevent-calculator]). Very high-risk CKD was characterized by either Stage G4 or G5 CKD (GFR < 30 mL/min/1.73 m²) or a classification of very high risk based on KDIGO guidelines, determined by GFR and urinary albumin-to-creatinine ratio ^[1]^. |
| CKM Stage4 | - Participants with self-reported established CVD, including coronary heart disease, angina, myocardial infarction, heart failure, and stroke. Atrial fibrillation and peripheral artery disease were not included due to data unavailability. |

Cardiovascular-kidney-metabolic (CKM) syndrome stages were classified using data from NHANES 2007-2018, according to the 2023 AHA Presidential Advisory on CKM Health ^[3]^. Definitions were adapted based on available NHANES data.

**reference:**

1.KDIGO 2021 Clinical Practice Guideline for the Management of Glomerular Diseases. Kidney international 2021, 100(4s): S1-s276.

2.Khan SS, Matsushita K, Sang Y, Ballew SH, Grams ME, Surapaneni A*, et al.* Development and Validation of the American Heart Association's PREVENT Equations. Circulation 2024, 149(6): 430-449.

3.Ndumele CE, Neeland IJ, Tuttle KR, Chow SL, Mathew RO, Khan SS*, et al.* A Synopsis of the Evidence for the Science and Clinical Management of Cardiovascular-Kidney-Metabolic (CKM) Syndrome: A Scientific Statement From the American Heart Association. Circulation 2023, 148(20): 1636-1664.

**Table S2**: Characteristics of participants stratified by CKM Syndrome Stages

| Characteristics | Total | Advanced CKM syndrome (Stages 3 or 4) | | P value |
| --- | --- | --- | --- | --- |
|  |  | (No = 9178) | Yes (n = 1895) |  |
| Age | 48.00(34.00,61.00) | 45.00(32.00,57.00) | 70.00(60.00,79.00) | < 0.0001 |
| Sex |  |  |  | < 0.001 |
| Male | 5233(47.25) | 4142(46.15) | 1091(54.62) |  |
| Female | 5840(52.75) | 5036(53.85) | 804(45.38) |  |
| Race |  |  |  | < 0.0001 |
| White | 4863(67.25) | 3802(66.16) | 1061(74.51) |  |
| Black | 2158(10.16) | 1768(9.97) | 390(11.38) |  |
| Mexican American | 1641(8.43) | 1463(8.95) | 178(4.89) |  |
| Other Hispanic | 1193(5.93) | 1034(6.30) | 159(3.50) |  |
| Other | 1218(8.24) | 1111(8.62) | 107(5.72) |  |
| Education levels |  |  |  | < 0.0001 |
| Less than high school | 1029(5.09) | 767(4.43) | 262(9.49) |  |
| High school or equivalent | 3969(32.77) | 3152(31.50) | 817(41.30) |  |
| College or above | 6075(62.14) | 5259(64.07) | 816(49.21) |  |
| BMI (kg/m2) | 27.90(24.10,32.60) | 27.76(24.00,32.40) | 28.59(25.10,33.70) | < 0.001 |
| BMI (kg/m2) |  |  |  | < 0.001 |
| Normal | 3135(30.50) | 2707(31.49) | 428(24.34) |  |
| Obesity | 4281(37.49) | 3472(36.72) | 809(43.15) |  |
| Overweight | 3632(31.82) | 2985(31.79) | 647(32.51) |  |
| Scr (mg/dL) | 0.83(0.71,0.98) | 0.82(0.70,0.95) | 0.97(0.82,1.17) | < 0.0001 |
| HbA1c | 5.50(5.20,5.80) | 5.40(5.20,5.70) | 5.80(5.50,6.50) | < 0.0001 |
| TC (mg/dL) | 189.00(164.00,217.00) | 191.00(166.00,218.00) | 175.00(150.00,204.00) | < 0.0001 |
| HDL-C(mg/dL) | 52.00(43.00,63.00) | 52.00(43.00,63.00) | 48.00(41.00,60.00) | < 0.0001 |
| UACR (mg/g) | 6.50(4.29,11.80) | 6.15(4.19,10.53) | 11.63(5.98,32.52) | < 0.0001 |
| eGFR (ml/min/1.73m^2^) | 98.09(83.62,110.73) | 100.38(87.57,112.44) | 75.99(59.10, 91.12) | < 0.0001 |
| SBP (mmHg) | 119.00(110.00,130.00) | 118.00(109.00,128.00) | 130.00(116.00,146.00) | < 0.0001 |
| DBP (mmHg) | 70.00(63.00,77.00) | 70.00(63.00,77.00) | 66.00(58.00,75.00) | < 0.0001 |
| Hypertension |  |  |  | < 0.0001 |
| No | 6285(61.50) | 5890(67.11) | 395(23.94) |  |
| Yes | 4788(38.50) | 3288(32.89) | 1500(76.06) |  |
| DM |  |  |  | < 0.0001 |
| No | 8713(84.00) | 7768(88.07) | 945(56.67) |  |
| Yes | 2360(16.00) | 1410(11.93) | 950(43.33) |  |
| Hyperlipidemia |  |  |  | < 0.0001 |
| No | 3004(28.50) | 2751(31.04) | 253(11.45) |  |
| Yes | 8069(71.50) | 6427(68.96) | 1642(88.55) |  |
| CVD |  |  |  | < 0.0001 |
| No | 9834(90.82) | 9177(100.00) | 657(29.28) |  |
| Yes | 1238(9.18) | 0(0.00) | 1238(70.72) |  |
| CKD prognosis |  |  |  | < 0.0001 |
| Low Risk | 9233(87.10) | 8232(91.41) | 1001(58.23) |  |
| Moderate Risk | 1273(9.39) | 810(7.33) | 463(23.18) |  |
| High Risk | 350(2.35) | 136(1.26) | 214(9.60) |  |
| Very High Risk | 217(1.17) | 0(0.00) | 217(8.99) |  |
| Mets |  |  |  | < 0.0001 |
| No | 6817(62.96) | 5878(65.27) | 939(47.48) |  |
| Yes | 4256(37.04) | 3300(34.73) | 956(52.52) |  |
| Energy intake (kcal/day) | 1973.50(1536.50,2500.50) | 2000.50(1562.00,2533.50) | 1799.50(1379.50,2267.00) | < 0.0001 |
| Alcohol intake (g/day) |  |  |  | < 0.001 |
| >30 | 881(9.71) | 793(10.14) | 88(7.13) |  |
| 0 | 7887(67.14) | 6383(66.21) | 1504(75.43) |  |
| 0.1-30 | 2258(22.75) | 1958(23.65) | 300(17.44) |  |
| Supplement use |  |  |  | < 0.0001 |
| No | 6139(54.23) | 5383(56.78) | 756(37.18) |  |
| Yes | 4934(45.77) | 3795(43.22) | 1139(62.82) |  |
| CDAI | 0.12(-1.97, 2.58) | 0.19(-1.87,2.72) | -0.55(-2.58,1.58) | < 0.0001 |
| Age | 48.00(34.00,61.00) | 45.00(32.00,57.00) | 70.00(60.00,79.00) | < 0.0001 |

BMI: Body Mass Index; Scr: Serum creatinine; TC: Total cholesterol; HDL-C: HDL cholesterol; UACR: Urine albumin to creatinine ratio; eGFR: estimated Glomerular Filtration Rate; SBP: Systolic Blood Pressure; DBP: Diastolic Blood Pressure; CVD: Cardiovascular Disease; CKD: Chronic Kidney Disease Prognosis; CDAI: Composite Dietary Antioxidant Index.

CDAI is a standardized composite index based on z-scores of six dietary antioxidants. Positive CDAI values indicate above-average total dietary antioxidant intake, while negative values indicate below-average intake relative to the population mean.
